# Identification of Trypanocidal Activity for Known Clinical Compounds Using a New *Trypanosoma cruzi* Hit-Discovery Screening Cascade

**DOI:** 10.1371/journal.pntd.0004584

**Published:** 2016-04-15

**Authors:** Manu De Rycker, John Thomas, Jennifer Riley, Stephen J. Brough, Tim J. Miles, David W. Gray

**Affiliations:** 1 Drug Discovery Unit, Division of Biological Chemistry and Drug Discovery, University of Dundee, Dundee, United Kingdom; 2 GlaxoSmithKline, Biological Sciences, Medicines Research Centre, Stevenage, Hertfordshire, United Kingdom; 3 GlaxoSmithKline, Diseases of the Developing World, Parque Tecnologico de Madrid, Servero Ochoa, Madrid, Spain; Harvard School of Public Health, UNITED STATES

## Abstract

Chagas disease is a significant health problem in Latin America and the available treatments have significant issues in terms of toxicity and efficacy. There is thus an urgent need to develop new treatments either via a repurposing strategy or through the development of new chemical entities. A key first step is the identification of compounds with anti-*Trypanosoma cruzi* activity from compound libraries. Here we describe a hit discovery screening cascade designed to specifically identify hits that have the appropriate anti-parasitic properties to warrant further development. The cascade consists of a primary imaging-based assay followed by newly developed and appropriately scaled secondary assays to predict the cidality and rate-of-kill of the compounds. Finally, we incorporated a cytochrome P450 CYP51 biochemical assay to remove compounds that owe their phenotypic response to inhibition of this enzyme. We report the use of the cascade in profiling two small libraries containing clinically tested compounds and identify Clemastine, Azelastine, Ifenprodil, Ziprasidone and Clofibrate as molecules having appropriate profiles. Analysis of clinical derived pharmacokinetic and toxicity data indicates that none of these are appropriate for repurposing but they may represent suitable start points for further optimisation for the treatment of Chagas disease.

## Author Summary

Chagas disease is an important health problem in Latin America. The disease is caused by the parasite *Trypanosoma cruzi*, which is transmitted to people via insects of the Triatomine family. There are currently only two treatments available, Nifurtimox and Benznidazole. These have serious problems including poor efficacy, strain-dependent drug sensitivity, resistance and toxicity to the patients. There is thus a great need to find new drugs for this disease. The first step in a typical drug discovery project is to find compounds that kill the parasite by screening large amounts of compounds in the laboratory. To do this one requires assays in which the effect of the compounds on the parasites can be seen. In this paper we describe two assays that together try to identify compounds that kill *T*.*cruzi* parasites. Such compounds are good candidates for further development and may eventually become new drugs. We tested our assays against a library of compounds with known clinical activity and identified several interesting hits. As a great deal of data already exists for these compounds they could potentially be developed into new treatments much faster than completely new compounds.

## Introduction

Chagas disease affects approximately 7 to 8 million people in Latin America [1] and a recent study estimates the mortality rate at 2.78 deaths per 100,000 inhabitants in Brazil [2] resulting in more than 5000 deaths annually in this country alone. Currently the only drugs approved for treatment of Chagas disease are the nitrodrugs Benznidazole and Nifurtimox [3]. Both have significant side-effects and their efficacy for treatment of chronic Chagas disease is unclear [4,5]. In addition there is naturally occurring resistance to these compounds [6]. Several new drugs are being developed, mostly targeting the protease cruzipain and the cytochrome p450 enzyme CYP51 (ergosterol biosynthesis pathway) [7,8]. Questions are emerging regarding CYP51 as a target due to strain-dependent variability in efficacy studies [9], and the recently reported high failure rate in humans for posaconazole [10–12]. There is thus a need to develop Chagas drugs with novel mechanisms, improved efficacy and safety profiles [13,14].

The causative agent of Chagas disease is *Trypanosoma cruzi*. *T*. *cruzi* metacyclic trypomastigotes are delivered to humans by infected insects of the Reduviidae family [15] and enter the cytoplasm of a wide variety of human host cells where they transform into replicative amastigotes. These eventually destroy their host cells and spread throughout the body [16].

The target product profile for new Chagas drugs demands clinical efficacy equal to or greater than the current nitro drugs [11,17]. In this paper, we present a screening cascade that details existing and new assays that we have developed to find start points to address the DNDi target product profile. These involve a high content intracellular primary screen, a novel, high throughput cidal assay that can be adapted togive an initial estimation of rate of kill together with a biochemical CYP51 assay to exclude compounds that owe their phenotypic response to engaging this target.

The development of new drugs from new chemical starting points identified in large scale screens can take a very long time (>10 years [18,19]). A much faster route is the repurposing of compounds which already have clinical data associated with them as this allows much faster progression through the drug development stages [20,21]. We have applied our screening cascade to identify trypanocidal molecules in a library of 963 clinically tested compounds and identified several drugs that had significant effects on *T*. *cruzi* growth. Detailed analysis indicates that they may not represent good repurposing tools. However, their activity, together with good pharmacokinetic parameters makes them attractive start points for lead optimisation.

## Methods

### Reagents

Benznidazole, Nifurtimox, and formaldehyde were obtained from Sigma. Posaconazole was from Sequoia Research Products. Hoechst 33342 from Life Technologies.

### Cells

Vero cells (ECCAC 84113001) were screened for mycoplasma infection and maintained in MEM medium supplemented with Glutamax (Life Technologies) and 10% (v/v) foetal calf serum (FCS) at 37°C in presence of 5% CO_2_. *T*. *cruzi* parasites (Silvio X10/7 A1, a clonal line kindly provided by Susan Wyllie and Prof. Alan Fairlamb) were maintained in Vero cells. On a weekly basis emerged trypomastigotes were used to infect a new Vero monolayer at multiplicity of infection (MOI) 1.5.

### Library and compound handling

The compounds tested were obtained from the NIH (Clinical Collection) and from SelleckChem (FDA-approved drug library). Compounds were dispensed into black 384-well assay plates (Corning) by acoustic dispensing (LabCyte ECHO). The single point primary screen was carried out at two concentrations (5μM and 15μM). For potency determinations, ten-point one in three dilution curves were generated, with a top concentration of 50μM. Potencies are reported as pEC_50_ (-LOG(EC_50_[M])).

### *in vitro T*. *cruzi* assays

For the primary intracellular assay the infection conditions were chosen so that no trypomastigote egress occurs during the compound incubation time. First, Vero cells were infected overnight with tissue culture derived *T*. *cruzi* trypomastigotes in T225 tissue culture flasks (MOI 5). Next, any remaining free trypomastigotes were washed away with serum free MEM and the infected Vero cells were harvested by trypsinisation. The infected Vero cells were then plated into 384-well plates containing the compounds to be tested, at 4000 cells per well in MEM media with 1% FCS. After 72h incubation at 37°C in presence of 5% CO_2,_ the plates were fixed with 4% formaldehyde for 20 minutes at room temperature and stained with 5μg/ml Hoechst 33342. The plates were imaged on a Perkin Elmer Operetta high-content imaging system using a 20x objective. Images were analysed using the Columbus system (Perkin Elmer). The algorithm for the primary assay first identified the Vero nuclei followed by demarcation of the cytoplasm and identification of intracellular amastigotes. This algorithm reported mean number of parasites per Vero cell and total number of Vero cells. Data analysis was as described in [22]. Robust z-factor was calculated with the following formula:

$$1-\frac{\left(3*(1.4826*MAD[0\%\;inhibition\;(raw\;data)]))+(3*(1.4826*MAD[100\%\;inhibition\;(raw\;data)])\right)}{MEDIAN\;[0\%\;inhibition\;(raw\;data)]-MEDIAN\;[100\%\;inhibition\;(raw\;data)]}$$

with MAD = Mean Absolute Deviation. Hits from the primary screen were selected based on the following criteria: *T*. *cruzi* activity (percent inhibition) **>** (median *T*. *cruzi* percent inhibition + 3 x robust Standard Deviation) and Vero activity (percent inhibition) **<** (median percent Vero inhibition + 3 x robust Standard Deviation).

### Cell replication assessments

Cell replication was assessed under assay conditions following the protocol for the primary assay described above. At each time-point one 384-well plate was fixed as described above (4, 24, 48, 72, 96 & 120 h) and imaged. Image analysis was as above and total number of Vero cells and total number of amastigotes in the field of view were counted and plotted against time. The doubling time for the amastigotes was calculated using the data in the exponential part of the growth curve.

Comparison of replication of infected versus non-infected cells was carried out in the same way (time-points 4, 24, 48 & 72 h) but the image analysis algorithm was modified to count infected and non-infected cells separately.

Cell replication in non-dividing Vero cells was measured using the same protocol as above except that the Vero cells were gamma-irradiated (2000 RAD) before infection and infected cells were plated in 96-well plates at 5x10^4^ cells per well.

### Static-cidal assay

The secondary static-cidal assay uses the same protocol as the standard assay with the following changes: infection is done at MOI 10, and infected Vero cells are incubated 88h at 37°C in presence of 5% CO_2_ prior to trypsinisation and plating. Four hours after plating a control plate (no compounds) was fixed with formaldehyde as described above. Compound containing plates were fixed after incubation for 96 hours. Compounds are run as ten point dose response curves in triplicate. The algorithm for the secondary assay was similar to the primary assay but reported percent infected Vero cells instead of the mean number of parasites. Data was normalised using the following formula:

$$\%\;Inhibition=100-\frac{\left(Raw\;data)-(\%infected\;[50µM\;Nifurtimox\;at\;t=96h]\right)}{\left(\%infected\;at\;t=0h)-(\%infected\;[50µM\;Nifurtimox\;at\;t=96h]\right)}*100$$

so that compounds that reduce the level of infection over time show a positive result and compounds that allow an increase in infection levels show a negative result.

### Rate-of-kill assay

The rate-of-kill assay uses the same protocol as the static-cidal assay but plates are incubated for 24, 48, 72 and 96 hours prior to fixing. Percent infected data is plotted as a time-course for each compound at every concentration. The minimum cidal concentration (MCC) is defined as the lowest concentration of compound that clears at least half as many Vero cells of intracellular parasites as Nifurtimox (50μM) within the timeframe of the assay.

### CYP51 assay as described in [23].

H1-receptor binding studies: Inhibition of agonist-induced calcium flux was measured in CHO cells expressing the H1-receptor using the method described in [24].

## Results

### High-content assay design

**Fig 1:**
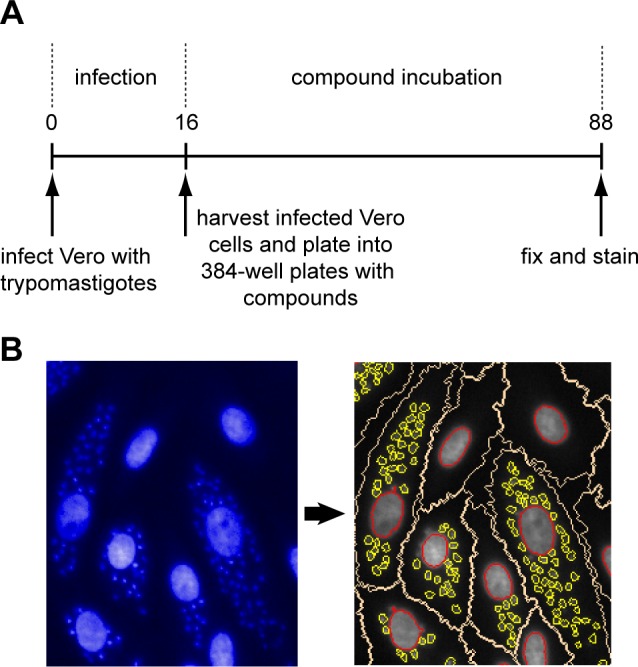
*T*. *cruzi* primary screening assay. Panel A. Schematic outline of the primary screening assay. Numbers are time in hours. Panel B. Image acquisition and analysis. Left-hand side shows an image as obtained from the high-content microscope. The large structures are the nuclei of the host cells and the small punctate structures are the nuclei of the amastigotes. The right-hand panel shows the segmentation carried out by the image analysis algorithm. The nuclei are delineated in red, the Vero cell cytoplasm in white and the amastigotes in yellow.

A high-content screening assay for intracellular *T*. *cruzi* amastigotes was developed based on previously published methods [25–27]. An outline of the assay is shown in Fig 1A and a detailed description of the assay can be found in the methods section. The only fluorescent marker used in this assay is a DNA stain. This allows usage of any *T*. *cruzi* strain and host cell line combination. Plates were imaged on an automated microscope and the images were analysed with an image analysis algorithm that we designed using Perkin Elmer Columbus (Fig 1B and methods). We determined the effect of DMSO on the assay and found that there was a stimulation of *T*. *cruzi* growth up to 0.5% DMSO followed by an increasingly detrimental effect at higher concentrations (Fig A in S1 Text). This profile was deemed compatible with compound screening and profiling as we use 0.5% DMSO as standard. Using the assay we determined the potency of several compounds with known anti-*T*. *cruzi* activity and found potencies that are in line with the literature [25,28,29] (Table 1).

**Table 1:** Potencies against intracellular *T*. *cruzi* for control compounds (N = 3).

Compound	Mean pEC_50_	StDev	EC_50_ (μM)
Posaconazole	9.0	0.1	0.001
Ketoconazole	8.7	0.1	0.002
Nifurtimox	6.6	0.04	0.26
Benznidazole	6.3	0.02	0.49

### Cell replication in the assay

**Fig 2:**
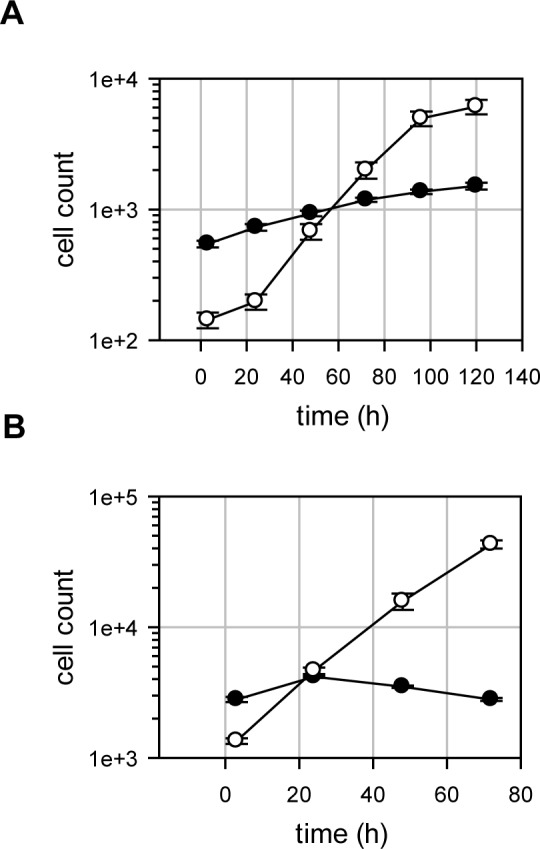
Replication of host cells and amastigotes. Panel A. A time-course was carried under assay conditions and plates were fixed every day followed by imaging and analysis. The full circles show the host cell counts and the open circles show the amastigote counts (n = 384 wells, error bars are standard deviations). The doubling time for the amastigotes was determined in the exponential growth window (between 24 and 96 h). Panel B. A time-course was carried out using gamma-irradiated Vero cells. Plates were fixed every day followed by imaging and analysis. The full circles show the host cell counts and the open circles show the amastigote counts (n = 18 wells from a single experiment, error bars are standard deviations).

To avoid a dilution effect of the *T*. *cruzi* amastigotes by Vero cell division we limited Vero cell division during the assay by reducing the amount of foetal calf serum from 10% to 1% and by plating the host cells near confluency. Under these conditions Vero cell division was measureable but minimal (*Td* = 85h) and the *T*. *cruzi* X10/7 A1 amastigote doubling time in exponential phase was 13.5h (Fig 2A). A separate experiment using irradiated Vero cells to completely block Vero cell replication yielded a *T*. *cruzi* amastigote doubling time of 11.2h (Fig 2B).

### Assay performance

To assess the suitability of the assay for high-throughput screening we determined standard screening statistics across 344 384-well plates (Table 2). The data show that the assay is robust, has low variability and a high signal to background. We achieved a throughput of 15,360 wells per batch while maintaining high-quality performance statistics. This allows the determination of 14,080 single point measurements (for library screening) or up to 1,280 potency determinations in a single run.

**Table 2:** Performance statistics of the *T*. *cruzi* assay.

Robust Z-factor	0.8 +/- 0.1 (n = 344)
Coefficient of Variation [%]	9.8 +/- 2.8 (n = 344)
Signal to Background	31 +/- 13 (n = 344)
Percent Infected Cells	27% (n≈1x10^6^)
Amastigotes / vero cell	6.8 +/- 3 (n = 344)
Throughput	40 x 384 well per batch

Values are averages +/- standard deviation, n is number of plates for Z-factor, %Coefficient of Variation, Signal:Background (S:B) and Amastigotes / vero cell (calculated from 32 control wells on each plate, includes both infected and non-infected cells) and number of Vero cells for Percent Infected Cells. Coefficient of Variance is for infected vehicle control wells. For S:B, signal is average number of amastigotes / Vero cell in vehicle control wells, background is average number of amastigotes / Vero cell in 100% effect (50μM Nifurtimox) control. Percent Infected cells and Amastigotes / Vero cell were determined at 72h.

### Static-cidal assay

**Fig 3:**
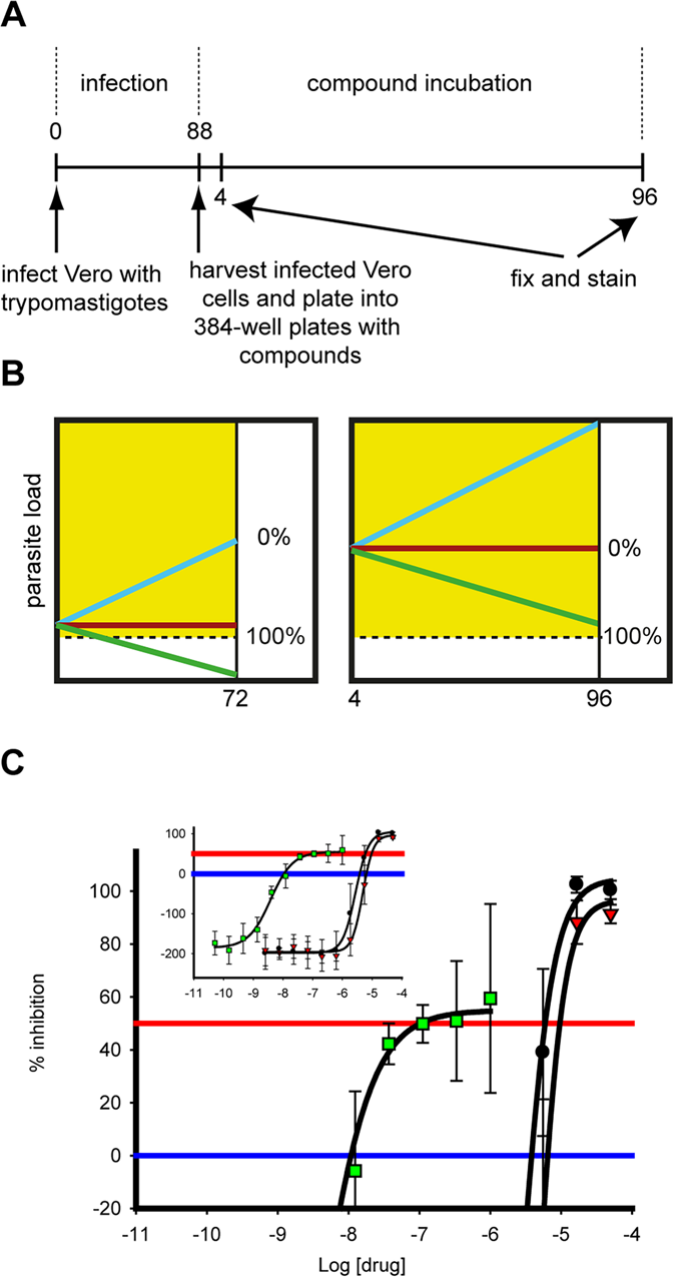
Static-cidal assay. Panel A. Schematic outline of the static-cidal screening assay. Numbers are time in hours. Times above the timeline are from addition of trypomastigotes, times below the line are starting from addition of the infected cells to the plates containing compounds. Panel B. Schematic outlining the differences between the primary (left) and the static-cidal (right) assays. The blue line represents an untreated sample, the red line a sample treated with a static acting compound and the green line a cidal compound. The x-axis is time (h) and the y-axis parasite load (amastigotes per Vero cell for the screening assay, percent infected Vero cells for the static-cidal assay). These charts do not represent data, they are models to explain the why there was a need to develop the static-cidal assay. The dashed line represents the detection limit of the assay. The locations of the 0% and 100% controls as used for data normalisation and curve fitting are shown. Panel C. Static-cidal assay dose-response curves for nifurtimox (black circles), benznidazole (red triangles) and posaconazole (green squares) x-axis shows concentration of compounds, y-axis percent inhibition normalised to starting level of infection (0% effect) and level of infection at 96h in presence of 50μM nifurtimox (100% effect).

To assess screening hits for their ability to be cytocidal rather than cytostatic or growth slowing we developed a medium throughput (400 compounds / batch) assay to predict cidality of compounds against intracellular *T*. *cruzi*. This assay is similar to the primary assay described above, but has an increased parasite load in the cells resulting from extending the time after infection before compound exposure and the use of a higher MOI (Fig 3A). The reason for increasing the parasite load is that in the primary assay we only see a few parasites (typically ≤2) in each infected cell after overnight infection. This low level of infection means that it is difficult to distinguish static from cidal compounds at the end of the assay (Fig 3B left panel). As shown in Fig 3B right panel, increasing the parasite load (to ~15 amastigotes / cell) increases the window between the detection limit of the assay and the starting burden and therefore allows much better separation between static and cidal compounds. We could not use the same image analysis approach as used for the primary assay as the number of parasites per cell becomes too high to segment the amastigotes reliably. Instead we developed an algorithm that assesses whether a Vero cell is infected or not, and we used the percentage of infected cells as primary measurement for the static-cidal assay. In order to readily identify cidal compounds we normalised the raw data using the starting level of infection as 0% effect and the final level of infection in presence of 50μM Nifurtimox as 100% effect control. As a result, compounds which do not change the level of infection give a maximum percent inhibition (MAX PI) of 0, compounds that allow progression of infection give a negative MAX PI and compounds that are likely to be cidal give a positive MAX PI. The results from this assay for nifurtimox, benznidazole and posaconazole are shown in Fig 3C and Table 3. MAX PI for both nifurtimox and benznidazole is around 100% inhibition, while it only reaches 60% for posaconazole in the 96h timeframe of the assay.

**Table 3:** Static-cidal (SC), Rate-of-kill (RoK) and CYP51 results for control compounds and selected hits from the bio-actives screen.

Compound	MAX PI (SC)^1^	pMCC (RoK)	CYP51 pIC50
Nifurtimox	103	5.3	< 5
Benznidazole	91	4.8	< 5
Posaconazole	59	7.0	7.5
Ketoconazole	61	8.1	7.9
Clotrimazole	60	7.6	7.4
Bifonazole	69	7.2	7.4
Itraconazole	71	7.2	7.5
Econazole	63	7.2	7.6
Voriconazole	70	7.2	7.4
Clemastine	108	6.7	< 5
Azelastine	106	5.7	< 5
Amlodipine	113	5.7	< 5
Ifenprodil	117	5.7	< 5
Ziprasidone	111	5.3	< 5
Clofibrate	116	4.8	< 5
Abiraterone	60	4.3	<6
Nilotinib	38	N/A	6.8

^1^ Maximum Percent Inhibition in the static cidal assay, only derived from compound concentrations that are not toxic to the host cells.

**Fig 4:**
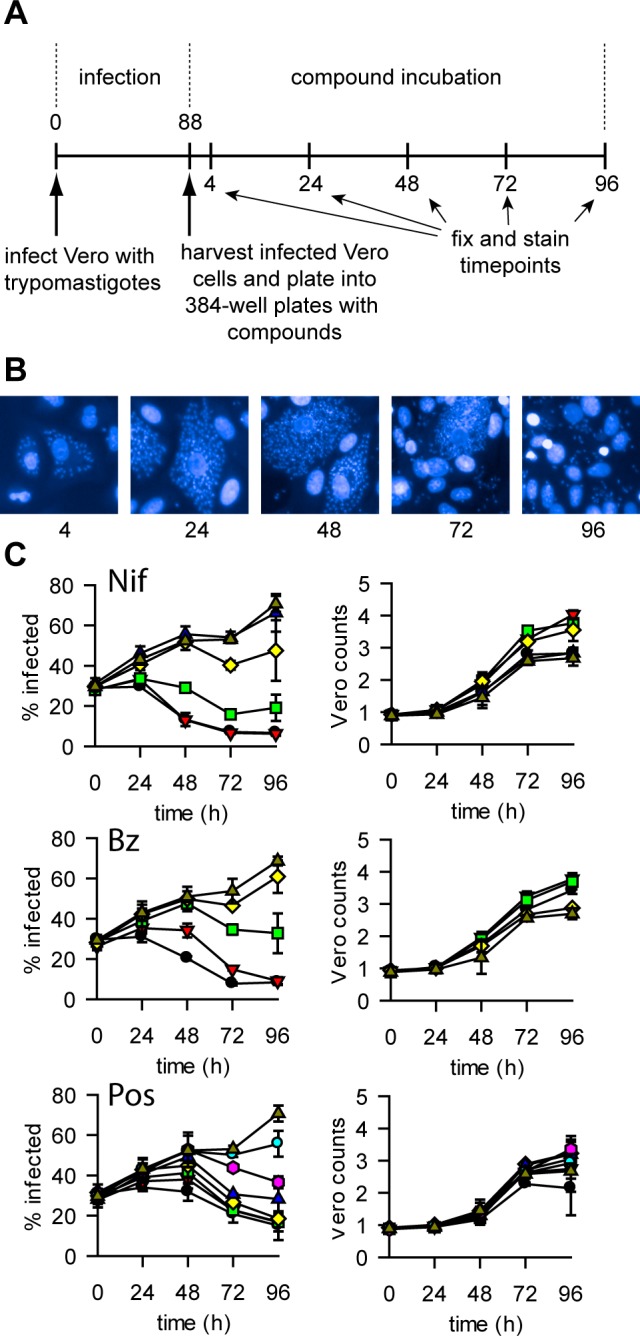
*T*. *cruzi* rate-of-kill assay. Panel A. Outline of the rate-of-kill assay. Numbers are time in hours. Times above the timeline are from addition of the trypomastigotes, times below the line are starting from addition of the infected cells to the plates containing compounds. Panel B. Representative images from untreated wells in the static-cidal assay at the different time-points. Panel C. Rate-of-kill profiles. Left panel shows percent infected cells against time, right panel shows Vero counts against time (n = 3, error bars = standard deviation) for Nifurtimox (top), Benznidazole (middle) and Posaconazole (bottom). Concentrations are as follows (μM) (all compounds were tested at the same concentrations except for Posaconazole, its concentrations are shown between brackets): black circle 50 (1), red triangle 17 (0.3), green square 5.6 (0.1), yellow diamond 1.9 (0.04), blue triangle 0.6 (0.01), pink hexagon 0.2 (0.004), cyan circle 0.07 (0.001), triangle dark yellow 0.

To obtain rate-of-kill information we carried out the static-cidal assay in timecourse format (Fig 4A), with timepoints taken every 24h. Due to the high level of infection at the start of the assay a cycle of trypomastigote release (and Vero lysis) occurs during the timecourse. The resulting re-infection leads to a significant increase in the percentage of infected cells during the course of the experiment (Fig 4B and 4C). Fig 4C shows the results of the rate-of-kill assay for nifurtimox, benznidazole and posaconazole. Nifurtimox and benznidazole exhibit similar kill profiles, with killing seen from 24h onwards and a final residual level of infection of ~8%. While posaconazole was much more potent in terms of EC_50_, it appears to be slower acting with a later onset of cidal activity and a higher level of infection at the end of the assay (~15%). We calculated the minimum cidal concentration (MCC) as the lowest concentration tested that resulted in a significant decrease in percentage of infected cells (see materials and methods). The pMCCs (-LOG(MCC[M]) for the control compounds are shown in Table 3. To confirm that a reduction in the percentage of infected cells was not merely due to a lower replication rate of infected Vero cells relative to non-infected Vero cells, we assessed replication of both types of cells and found that infected cells divide as well as or better than non-infected cells (Fig B in S1 Text).

**Fig 5:**
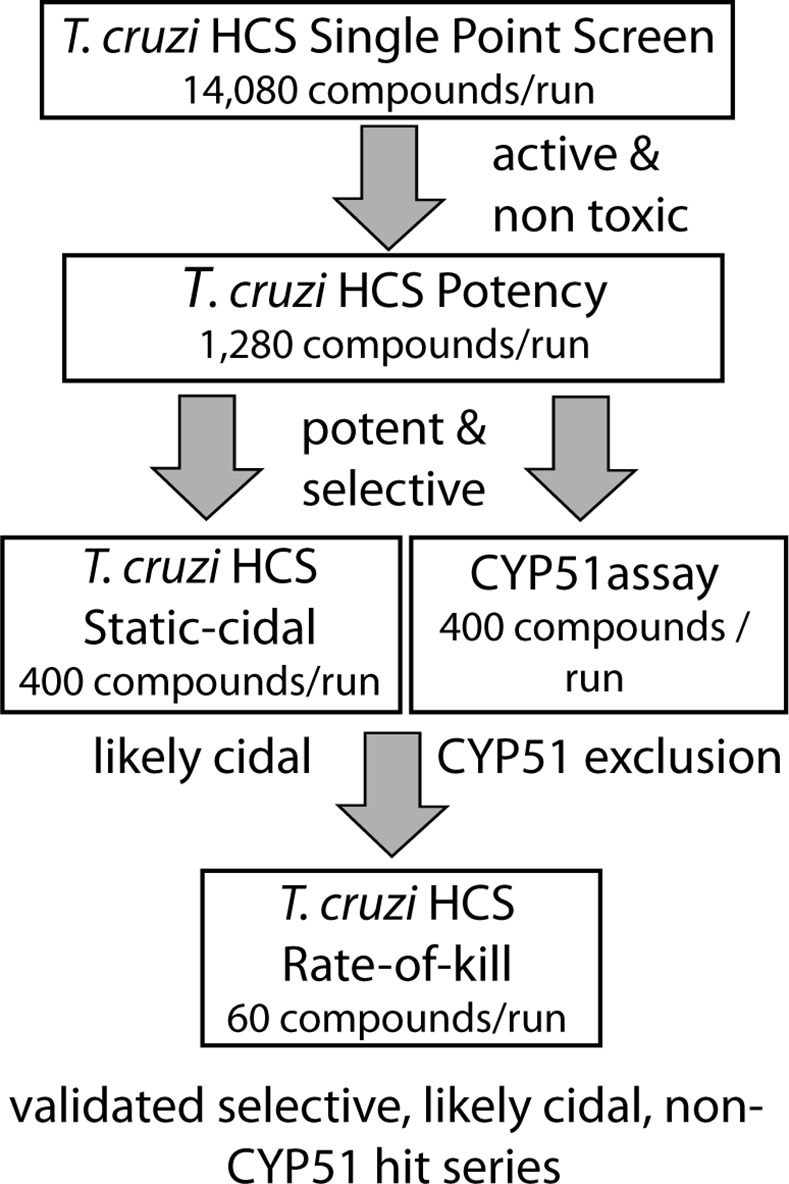
*T*.*cruzi* screening cascade. Schematic of *T*.*cruzi* screening cascade. Primary assay is the high-content intracellular amastigote assay at a single concentration. Compounds that achieve a set level of inhibition of amastigotes and show no toxicity against the host cells are progressed to potency determination in the same assay (using 10 compound concentrations). A potency and selectivity cut-off are next applied (cut-offs vary based on library used, MW of compounds, etc.) and the most promising compounds are progressed to the static-cidal assay to predict cidality and to the CYP51 assay to identify any compounds that may act through this target. Compounds that are predicted to be cidal and CYP51 independent are further progressed into the rate-of-kill assay and eventually into a hits-to-lead programme.

Together the assays described above constitute our *in vitro* screening cascade for *T*.*cruzi* (Fig 5). The cascade is tailored so that the initial assays provide the high throughput required for library screening, while the secondary assays have lower, but sufficient throughput to give key information regarding cidality and rate-of-kill for the hit compounds from the primary screen. The cascade also includes a biochemical CYP51 assay to assess whether compounds act through this mode of action.

### Bio-actives library screen

**Fig 6:**
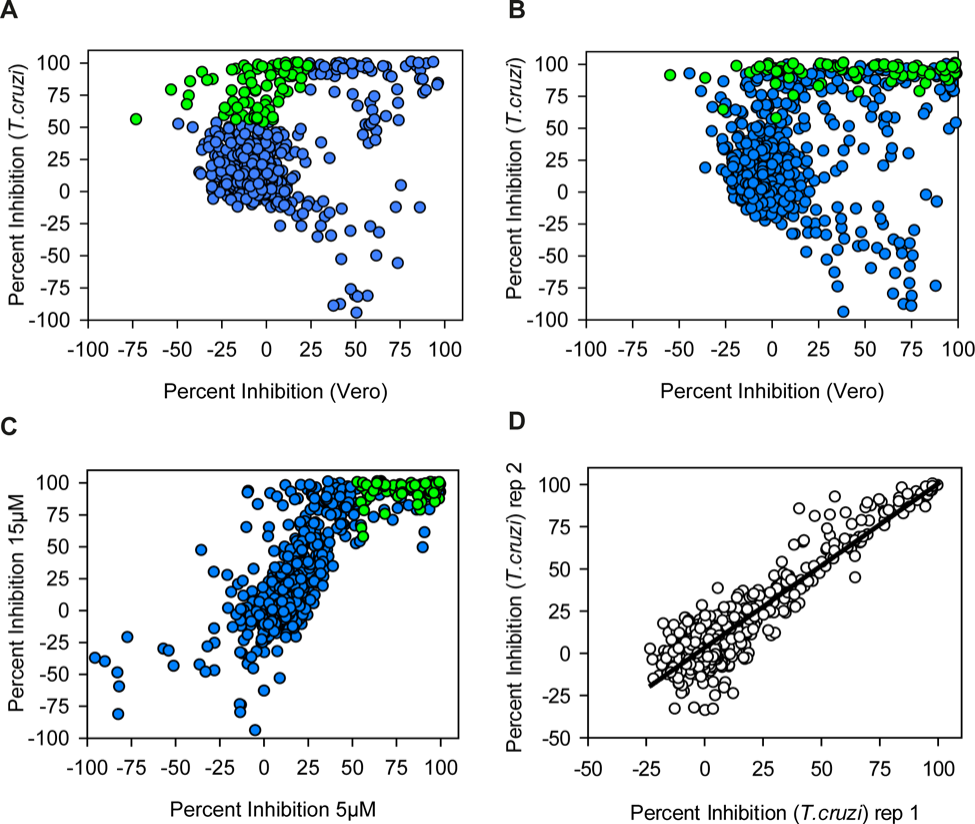
Results of clinical collection compound primary screen. Panel A. Scatterplot representing the results for the screen at 5 μM. Green circles represent the hits, blue circles are all the other compounds. Panel B. Scatterplot representing the results for the screen at 15 μM. Green circles represent the hits from the 5 μM screen, blue circles are all the other compounds. Panel C. Scatterplot comparing *T*. *cruzi* percent inhibition between 5 μM and 15 μM screens. Panel D. Replicates plot for a subset of compounds screened twice at 5 μM (n = 579, R^2^ = 0.86).

**Fig 7:**
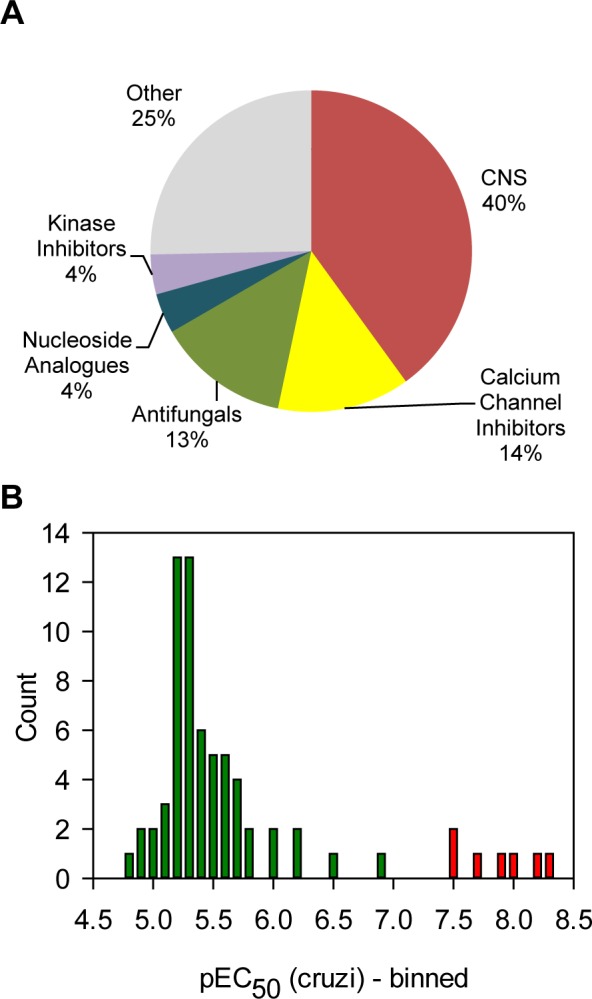
Analysis of the hits. Panel A. All the hits from the 5 μM screen were annotated with a general pharmaceutical class. The chart shows the frequency for each class. Panel B. The potencies for all the hits were determined and represented as a frequency table. Bars in red represent CYP51 inhibitors, green = all other classes).

We used the screening cascade to profile two sets of compounds that have known bio-activity and associated clinical data. The SelleckChem set contains 421 FDA-approved drugs and the NIH Clinical Collection set consists of 727 compounds that have been used in clinical trials. These libraries were tested in single replicate at 5 and 15 μM. Fig 6 panels A&B show the results for both screens. As expected there were substantially more compounds with toxicity towards the Vero cells at 15 μM (20% of all compounds) then at 5 μM (11%) (using an arbitrary Vero percent inhibition cut-off of 30%). Panel C shows the relationship between *T*. *cruzi* inhibition at 5 and 15 μM and shows that there was good correlation between both screens with an expected shift to higher activity at 15 μM. We also repeated a set of compounds at 5 μM to assess reproducibility, and as shown in panel D there was a good correlation between the two independent replicates (R^2^ = 0.86). In order to select hits for follow-up we set criteria for both *T*. *cruzi* activity and Vero toxicity. Hits were defined as causing a statistically significant reduction of *T*. *cruzi* amastigotes with no statistically relevant toxicity (further defined in Methods section). This selection procedure yielded 75 hits, which are marked green on Fig 6A. Some compounds appeared more than once because they were present in both compound libraries. After removing the duplicates there were 69 hits left. Grouping of the hits by target area showed that a large subset of the hit compounds target the CNS (40%), followed by calcium channel inhibitors and antifungals (Fig 7A). We next carried out 10-point dose-response curves on the hits to determine the *T*. *cruzi* and Vero cell pEC_50_s (Table A in S1 Text). Fig 7B shows the distribution of potencies found for the hit compounds. Most compounds only had moderate activity with the mode of the distribution being pEC_50_ 5.3 (EC_50_ = 5.6 μM). A small subset of compounds had much better activity, with seven compounds showing a pEC_50_ > 7. All these compounds are azoles known to inhibit the enzyme lanosterol-14-demethylase (CYP51) [30,31].

**Fig 8:**
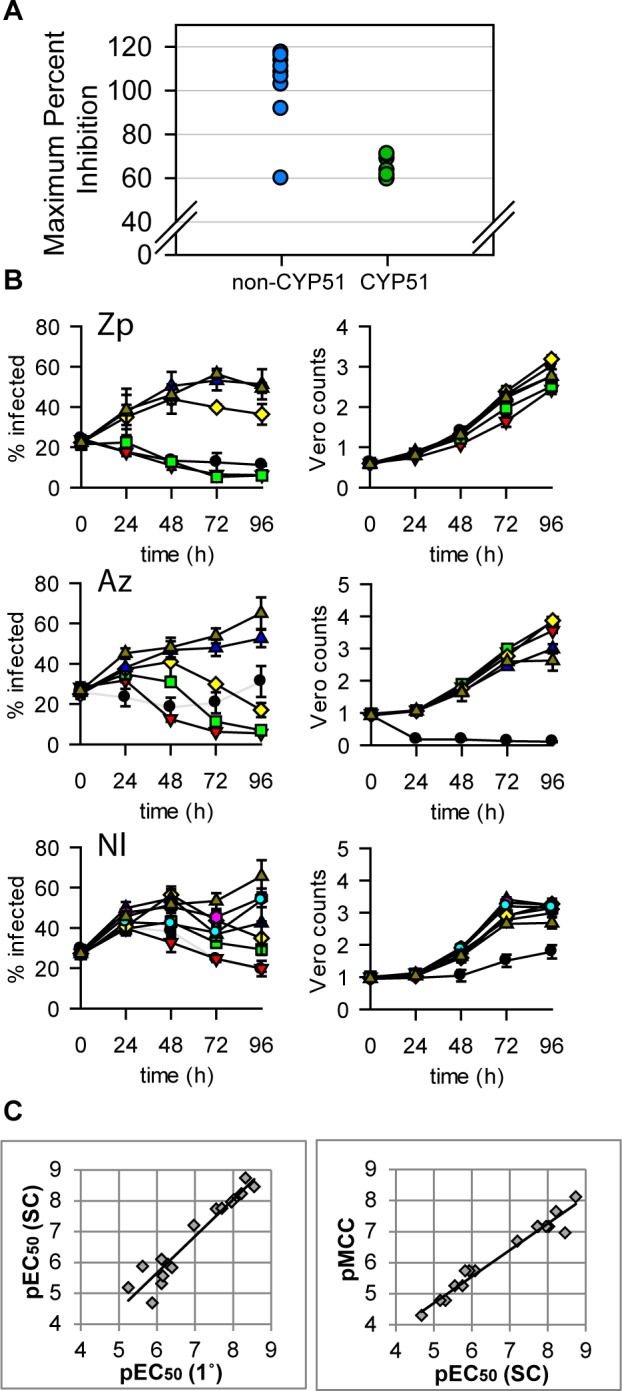
Static-cidal results for hits from the screen. Panel A. Maximum percent inhibition seen in static-cidal assay relative to Nifurtimox at t = 96h (100%) and t = 0h (0%). The data-set is split in half; the CYP51 inhibitors are marked in green, the other compounds in blue. Panel B. Static-cidal profiles for 3 of the hits: Ziprasidone (Zp), Azelastine (Az) and Nilotinib (Nl). Infection curves at concentrations that were toxic to the host cells are shown in light grey instead of black. Concentrations are as follows (μM): black circle 50, red triangle 17, green square 5.6, yellow diamond 1.9, blue triangle 0.6, pink hexagon 0.2, cyan circle 0.07, triangle dark yellow 0. Panel C. Left panel: scatterplot comparing the potencies obtained in the primary screening assay (x-axis) with the potencies obtained in the static-cidal assay (y-axis). A linear regression was fitted to the data (R^2^ = 0.92). Right panel: scatterplot comparing the potencies obtained in the static-cidal assay (x-axis) with the minimum cidal concentration (y-axis) obtained in the rate-of-kill assay. A linear regression was fitted to the data (R^2^ = 0.96). Panel C:

We next assessed selected potent and selective hits from our screen in the static-cidal and CYP51 assays (Table 3). In the static-cidal assay we observed clear differences in maximum inhibition ranging from 38% for nilotinib to 117% for ifenprodil. As expected, the azoles showed strong CYP51 inhibition as did nilotinib. While the CYP51 azoles showed high potency in the primary assays we found that in the static-cidal assay the maximum level of activity achieved was poor compared to most other compounds tested (Fig 8A). Rate-of-kill profiles were also determined and examples are shown in Fig 8B; ziprasidone showed fast onset of cidality, azelastine a more delayed response and nilotinib a much slower rate (this compound did not reach the threshold for cidality over the timecourse of the assay). All the CYP51 inhibitors showed a similar profile to posaconazole. As the static-cidal and rate-of-kill assays are carried out at 10 concentrations for each compound we could use the data to determine EC_50_s and MCCs and compared them to the results from the primary assay. We found that the EC_50_s from the primary and static-cidal assays were similar (Fig 6C left panel). Fig 6C right panel shows that the rate-of-kill assay MCC and static-cidal EC_50_ also correlate, with the MCC on average 0.6 LOG units lower than the EC_50_.

### Azelastine

One of the compounds with an interesting profile was azelastine, an antihistamine that acts through inhibition of the histamine H1 receptor [32]. As a chemotype, these have been previously described as having trypanocidal activity [25,33]. To assess if the antitrypanosomal activity of this compound can be separated from its H1 activity we assessed the H1 and *T*. *cruzi* activity for a set of azelastine analogues (Fig C and Table B in S1 Text) and found that H1 binding could be significantly reduced (>1000-fold) while maintaining *T*.*cruzi* activity.

## Discussion

In order to find start points for drug discovery a suite of assays is required that combine disease relevance and scale to allow the iterative deselection of chemotypes with inappropriate properties. Here we describe the use of a *T*. *cruzi* screening cascade to identify new repurposing candidates for the treatment of Chagas disease. The cascade consists of a single-point primary screen, followed by potency determination, a cidality and rate-of-kill assessment and *in vitro* CYP51 activity assessment (Fig 5).

Various *in vitro* assays have been used as a primary screen to identify compounds that kill *T*. *cruzi*. Axenically grown epimastigotes as a surrogate for the disease relevant stages of the disease have been developed as they are more amenable to high-throughput screening [34–36]. However, using the insect stage of *T*. *cruzi* increases the risk of not assessing the relevant biology. With that in mind there has been a recent transition to assaying intracellular parasites using reporter systems and high-content screening methods [25,26,37–43].

Our primary screening assay is distinct from previously published *T*. *cruzi* high-content assays in that we infect the Vero cells in bulk and then plate infected Vero cells into 384-well plates containing compounds [25,26,38,42]. This is important in terms of streamlining the screening process and allows us to run this assay at relatively high throughput (~14,000 wells / run). These changes have not affected the results produced by the assay as the potencies that we determined for standard compounds and for the screening hits align well with previously published results (see below).

Our previous experience in finding startpoints for kinetoplastid drug discovery has highlighted the issue of separating growth-slowing from cytocidal compounds [44,45]. We have observed significant replication of the *T*. *cruzi* amastigotes in the assay described here (Fig 2) which contrasts with our intracellular *Leishmania* assay, where we see almost no replication [22]. This combined with a low level of infection means that our *T*. *cruzi* high-throughput assay is poor at distinguishing static from cidal compounds (Fig 3B). We have therefore developed a secondary assay to assess whether compounds act through a cidal mechanism or not (Fig 3). This assay is different from previously published cidality assays in that it is much shorter, has far higher throughput and does not include a washout step [46,47]. It represents a pragmatic answer to deselecting chemotypes with inappropriate mechanisms of action early and efficiently. By limiting compound numbers but taking more time-points, the assay can be configured to give an indication of rate of kill. While we currently don’t have enough data to assess how cidality and rate-of-kill relate to *in vivo* efficacy, from a pragmatic point of view we prioritise predicted cidal compounds over static ones and fast acting over slower acting which is in line with compound progression criteria proposed by the Drugs for Neglected Diseases initiative (DND*i*) [11]. We have previously shown that a similar static-cidal assay for *Trypanosoma brucei* provides superior predicting power of *in vivo* efficacy in an animal model compared to the primary assay [48]. However, as our static-cidal assay only gives a prediction regarding the cidal nature of a compound, further verification of the most interesting series in a low throughput washout experiments will be valuable.

A key measurement returned by the static-cidal assay is the minimum cidal concentration (MCC). This is the lowest concentration of compound necessary to see cidal activity in the assay. We observed that around 3-fold more compound is required to reach the MCC compared to the EC_50_. This is not surprising as one would expect to see cidal activity near the EC_99_ of a compound rather than the EC_50_.

We employed the above screening cascade to screen two libraries containing molecules with known clinical data with an eye on identifying candidates for repurposing towards the treatment of Chagas disease. We carried out this screen at two concentrations (5 and 15 μM) and the comparison between these two runs exemplifies the difficulty of choosing a suitable screening concentration when working with intracellular parasites. A higher concentration increases the hit-rate and allows finding less potent hits, but also increases the risk of toxicity towards the host cell which would hide any potential anti-parasitic activity at lower concentrations (Fig 6C).

The potency of most hits proved to be modest, with the exception of the CYP51 inhibitors which were very potent (Fig 7B). We found 11 hits that were previously shown to have activity against *T*. *cruzi* and the potencies we measured correlated well with the published values (R^2^ = 0.7)[25].

In spite of their high potency, the CYP51 inhibitors all exhibited a moderately slow killing profile resulting in a relatively high number of infected cells remaining at the end of the static-cidal assay timecourse (reflected by poor level of inhibition, Fig 8A). The discrepancy between potency and rate-of-kill is not unexpected as potency reflects the affinity for the target(s) and the concentration of the compound inside the amastigote whereas the rate-of-kill reflects the mode-of-action (MOA) of the compound. Targeting some MOAs will result in fast, replication independent cell death (membrane integrity, non-specific toxicity, etc) whereas others will require a certain amount of cell division before the cells start dying (enzymes in biosynthetic pathways, slow turnover targets, etc). Our results suggest that the CYP51 inhibitors fall in the second category while the nitrodrugs fall in the first one. Whether the poor performance of the CYP51 inhibitors in *T*. *cruzi* clinical trials is related to this or to other factors (e.g. pharmacokinetics) remains to be ascertained.

We classified the remaining hits from the screen and found that many are CNS targeting lipophilic amines. As the targets for such drugs do not exist in *T*. *cruzi* amastigotes or Vero cells these are likely to be off-target driven. This category of molecules is known to be particularly promiscuous [49]. Because of their low therapeutic dosage and potential side-effects these may not be a progressable family of hits. More interesting is the observation that many calcium channel blockers were identified. This supports the idea that interfering with Ca^2+^ homeostasis may be a good approach for *T*. *cruzi* chemotherapy [50]. In addition we identified many CYP51 inhibitors which were already known to have anti-*T*. *cruzi* activity.

In this report we have identified several known clinical compounds as candidates for a repurposing strategy for Chagas disease, some of which were also identified in independent screening efforts [25,33,51]. Based on the profiles shown (potency, selectivity and cidality) the most promising drugs are clemastine, azelastine, ifenprodil, ziprasidone and clofibrate. Pharmacokinetic data in humans exists for these molecules [32,52–55]. They all have reasonable oral bioavailability but the free blood concentrations achievable are currently limited by toxicity, normally associated with their primary human target. While this means that these molecules are not suitable for repurposing, the H1 antagonists in particular look very attractive as start points for optimisation. Either removing the H1 antagonism using the large quantity of data on the H1 pharmacophore or eliminating CNS penetration would give the potential for developing effective Chagas treatments. We tested a panel of azelastine analogues and show that the *T*.*cruzi* activity can indeed be separated from H1 antagonism (Fig C in S1 Text).

In conclusion, the novel screening cascade for Chagas hit-discovery described here allows high-throughput screening of large compound collections through a robust high-content intracellular assay. In a second step, non-cidal compound classes are removed at a lower, but still significant, throughput. In addition the cascade includes a CYP51 assay to assess whether the compounds act through this mode-of-action. The combination of these assays allows selection of the most promising chemotypes for further development.

## Supporting Information

S1 Text Fig A–DMSO tolerance on Vero and amastigote/Vero cell ratio. Fig B–Effect of infection on Vero cell replication. Fig C-Plot of T. cruzi potency against human H1 binding affinity for a panel of azelastine analogues. Table A–Identities, structures and potencies of hits from the bioactives screen. Table B -Identity and structure of azelastine analogues.
(DOCX)
