# Supplementary material for: Identification of Trypanocidal Activity for Known Clinical Compounds Using a New Trypanosoma cruzi Hit-Discovery Screening Cascade
Source: PLoS Negl Trop Dis. 2016 Apr 15;10(4):e0004584. doi: 10.1371/journal.pntd.0004584 (PMC4833300; doi:10.1371/journal.pntd.0004584)
Supplement: S1 Text — Fig A–DMSO tolerance on Vero and amastigote/Vero cell ratio. Fig B–Effect of infection on Vero cell replication. Fig C-Plot of T. cruzi potency against human H1 binding affinity for a panel of azelastine analogues. Table A–Identities, structures and potencies of hits from the bioactives screen. Table B -Identity and structure of azelastine analogues. (DOCX) [file pntd.0004584.s001.docx]

Figure A. DMSO tolerance

Figure B. Effect of infection on replication

Table A. Identities and Potencies for the hits from the bio-actives screen.

| **Structure** | **ID** | **Name** | **pEC50 (cruzi)** | **pEC50 (Vero)** |
| --- | --- | --- | --- | --- |
|    \| C_26_H_28_Cl_2_N_4_O_4_ \| \| --- \| | DDD00089152 | Ketoconazole | 8.3 | 4.5 |
|    \| C_22_H_17_ClN_2_ \| \| --- \| | DDD00072003 | Clotrimazole | 8.2 | 4.9 |
|    \| C_22_H_18_N_2_ \| \| --- \| | DDD00203474 | Bifonazole | 8.0 | 4.8 |
|    \| C_35_H_38_Cl_2_N_8_O_4_ \| \| --- \| | DDD00771212 | Itraconazole | 8.0 | 4.8 |
|    \| C_18_H_15_Cl_3_N_2_O \| \| --- \| | DDD00071963 | Econazole | 7.7 | 5.0 |
|    \| C_16_H_14_F_3_N_5_O \| \| --- \| | DDD00203439 | Voriconazole | 7.6 | 4.3 |
|    \| C_18_H_14_Cl_4_N_2_O \| \| --- \| | DDD00072350 | Miconazole | 7.5 | 5.1 |
|    \| C_21_H_26_ClNO \| \| --- \| | DDD00089423 | Clemastine | 7.0 | 4.6 |
|    \| C_22_H_24_ClN_3_O \| \| --- \| | DDD00771290 | Azelastine | 6.6 | 4.8 |
|    \| C_20_H_25_ClN_2_O_5_ \| \| --- \| | DDD00089534 | Amlodipine | 6.3 | 5.1 |
|    \| C_21_H_27_NO_2_ \| \| --- \| | DDD00071948 | Ifenprodil | 6.3 | 4.3 |
|    \| C_26_H_33_NO_2_ \| \| --- \| | DDD00771727 | Abiraterone | 6.1 | 4.5 |
|    \| C_6_H_6_N_4_O_4_- \| \| --- \| | DDD00071930 | Nitrofurazone | 6.0 | 4.8 |
|    \| C_27_H_28_N_2_O_7_- \| \| --- \| | DDD00089508 | Cilnidipine | 5.9 | 4.8 |
|    \| C_21_H_27_NO \| \| --- \| | DDD00203415 | Benproperine | 5.9 | 4.8 |
|    \| C_21_H_21_ClN_4_OS \| \| --- \| | DDD00771620 | Ziprasidone | 5.8 | 4.6 |
|    \| C_21_H_26_N_2_O_7_- \| \| --- \| | DDD00203537 | Nimodipine | 5.7 | 4.8 |
|    \| C_28_H_31_N_3_O_6_- \| \| --- \| | DDD00203446 | Benidipine | 5.7 | 5.0 |
|    \| C_18_H_18_O_2_ \| \| --- \| | DDD00718048 | Honokiol | 5.7 | 5.1 |
|    \| C_26_H_37_NO_2_ \| \| --- \| | DDD00089160 | N-arachidonoylaminophenol | 5.7 | 4.8 |
|    \| C_28_H_30_N_6_OS \| \| --- \| | DDD00771571 | Masitinib | 5.7 | 5.1 |
|    \| C_10_H_13_N_5_O_3_ \| \| --- \| | DDD00237073 | 3'-deoxydenosine | 5.6 | 4.3 |
|    \| C_28_H_22_F_3_N_7_O \| \| --- \| | DDD00771565 | Nilotinib | 5.6 | 4.8 |
|    \| C_12_H_9_NS \| \| --- \| | DDD00728190 | Phenothiazine | 5.6 | 4.8 |
|    \| C_20_H_24_ClN_3_S \| \| --- \| | DDD00066604 | Prochlorperazine | 5.6 | 5.2 |
|    \| C_23_H_27_Cl_2_N_3_O_2_ \| \| --- \| | DDD00771276 | Aripiprazole | 5.6 | 4.9 |
|    \| C_21_H_26_N_2_S_2_ \| \| --- \| | DDD00072344 | Thioridazine | 5.6 | 5.2 |
|    \| C_30_H_32_N_2_O_2_ \| \| --- \| | DDD00203482 | Diphenoxylate | 5.6 | 4.8 |
|    \| C_18_H_23_NO \| \| --- \| | DDD00089258 | Bifemelane | 5.5 | 4.8 |
|    \| C_22_H_26_F_3_N_3_OS \| \| --- \| | DDD00071956 | Fluphenazine | 5.5 | 5.0 |
|    \| C_18_H_18_ClNS \| \| --- \| | DDD00771663 | Chlorprothixene | 5.5 | 4.8 |
|    \| C_17_H_17_F_3_N_4_ \| \| --- \| | DDD00771283 | CGS 12066B | 5.5 | 4.8 |
|    \| C_27_H_30_F_2_N_2_O_3_ \| \| --- \| | DDD00203486 | Lomerizine | 5.4 | 4.8 |
|    \| C_26_H_29_N_3_O_6_- \| \| --- \| | DDD00771347 | Cardene | 5.4 | 4.8 |
|    \| C_18_H_20_N_2_O_6_- \| \| --- \| | DDD00203472 | Nitrendipine | 5.4 | 4.8 |
|    \| C_33_H_40_N_2_O_9_ \| \| --- \| | DDD00089475 | Reserpine | 5.4 | 4.3 |
|    \| C_22_H_27_NO_2_ \| \| --- \| | DDD00771174 | Danazol | 5.4 | 4.8 |
|    \| C_12_H_15_N_5_O_3_ \| \| --- \| | DDD00771732 | Entecavir | 5.4 | 4.8 |
|    \| C_17_H_19_ClN_2_S \| \| --- \| | DDD00066605 | Chlorpromazine | 5.4 | 5.0 |
|    \| C_29_H_33_ClN_2_O_2_ \| \| --- \| | DDD00072189 | Loperamide | 5.4 | 4.7 |
|    \| C_32_H_47_F_5_O_3_S \| \| --- \| | DDD00771578 | Fulvestrant | 5.3 | 4.8 |
| C_19_H_20_FNO_3_ | DDD00072581 | Paroxetine | 5.3 | 4.9 |
|    \| C_21_H_23_NO \| \| --- \| | DDD00771673 | Dapoxetine | 5.3 | 4.8 |
|    \| C_14_H_16_N_2_O_2_ \| \| --- \| | DDD00089418 | Etomidate | 5.3 | 4.3 |
|    \| C_22_H_27_N_3_O_2_S_2_ \| \| --- \| | DDD00203735 | Thiothixene | 5.3 | 5.1 |
|    \| C_25_H_27_ClN_2_ \| \| --- \| | DDD00071813 | Meclizine | 5.3 | 4.7 |
|    \| C_23_H_25_N_5_O_5_ \| \| --- \| | DDD00072588 | Doxazosin | 5.3 | 4.8 |
|    \| C_24_H_26_N_2_O_4_ \| \| --- \| | DDD00089482 | Carvedilol | 5.3 | 4.8 |
|    \| C_18_H_19_Cl_2_NO_4_ \| \| --- \| | DDD00203741 | Felodipine | 5.3 | 4.8 |
|    \| C_29_H_31_N_7_O \| \| --- \| | DDD00771286 | Imatinib | 5.3 | 4.8 |
|    \| C_21_H_27_N_3_ \| \| --- \| | DDD00089407 | Rimcazole | 5.3 | 5.0 |
|    \| C_20_H_23_N \| \| --- \| | DDD00071714 | Maprotilline | 5.3 | 4.8 |
|    \| C_21_H_32_N_2_O \| \| --- \| | DDD00771210 | Stanozolol | 5.3 | 4.8 |
|    \| C_16_H_15_Cl_2_N \| \| --- \| | DDD00089028 | Indatraline | 5.3 | 4.9 |
|    \| C_19_H_21_N_5_O_4_ \| \| --- \| | DDD00072649 | Prazosin | 5.3 | 5.0 |
|    \| C_12_H_15_ClO_3_ \| \| --- \| | DDD00089746 | Clofibrate | 5.2 | 4.8 |
|    \| C_26_H_26_N_2_O_3_ \| \| --- \| | DDD00089440 | SDM25N | 5.2 | 4.8 |
|    \| C_33_H_34_N_6_O_6_ \| \| --- \| | DDD00771693 | Candesartan cilexetil | 5.2 | 4.9 |
|    \| C_17_H_18_F_3_NO \| \| --- \| | DDD00071907 | Fluoxetine | 5.2 | 4.7 |
|    \| C_23_H_30_N_4_O_2_S \| \| --- \| | DDD00771265 | Perospirone | 5.2 | 4.8 |
|    \| C_19_H_23_ClN_2_ \| \| --- \| | DDD00065535 | Clomipramine | 5.2 | 4.8 |
|    \| C_20_H_32_N_5_O_8_P \| \| --- \| | DDD00771651 | Adefovir Dipivoxil | 5.2 | 4.8 |
|    \| C_19_H_19_ClN_2_ \| \| --- \| | DDD00300268 | Desloratadine | 5.2 | 4.8 |
|    \| C_18_H_19_NOS \| \| --- \| | DDD00203552 | Duloxetine | 5.2 | 4.8 |
|    \| C_12_H_7_Cl_3_O_2_ \| \| --- \| | DDD00771322 | Triclosan | 5.1 | 5.0 |
|    \| C_21_H_35_NO \| \| --- \| | DDD00771642 | Amorolfine | 5.0 | 4.8 |
|    \| C_47_H_64_N_4_O_12_ \| \| --- \| | DDD00203506 | Rifapentine | 5.0 | 4.8 |
|    \| C_23_H_32_O_3_ \| \| --- \| | DDD00771182 | 17β-estradiol 17-valerate | 5.0 | 4.9 |
|    \| C_27_H_30_F_6_N_2_O_2_ \| \| --- \| | DDD00771580 | Dutasteride | 4.8 | 4.8 |

**Figure C *T.cruzi* and H1-binding for a panel of Azelastine analogues**

**
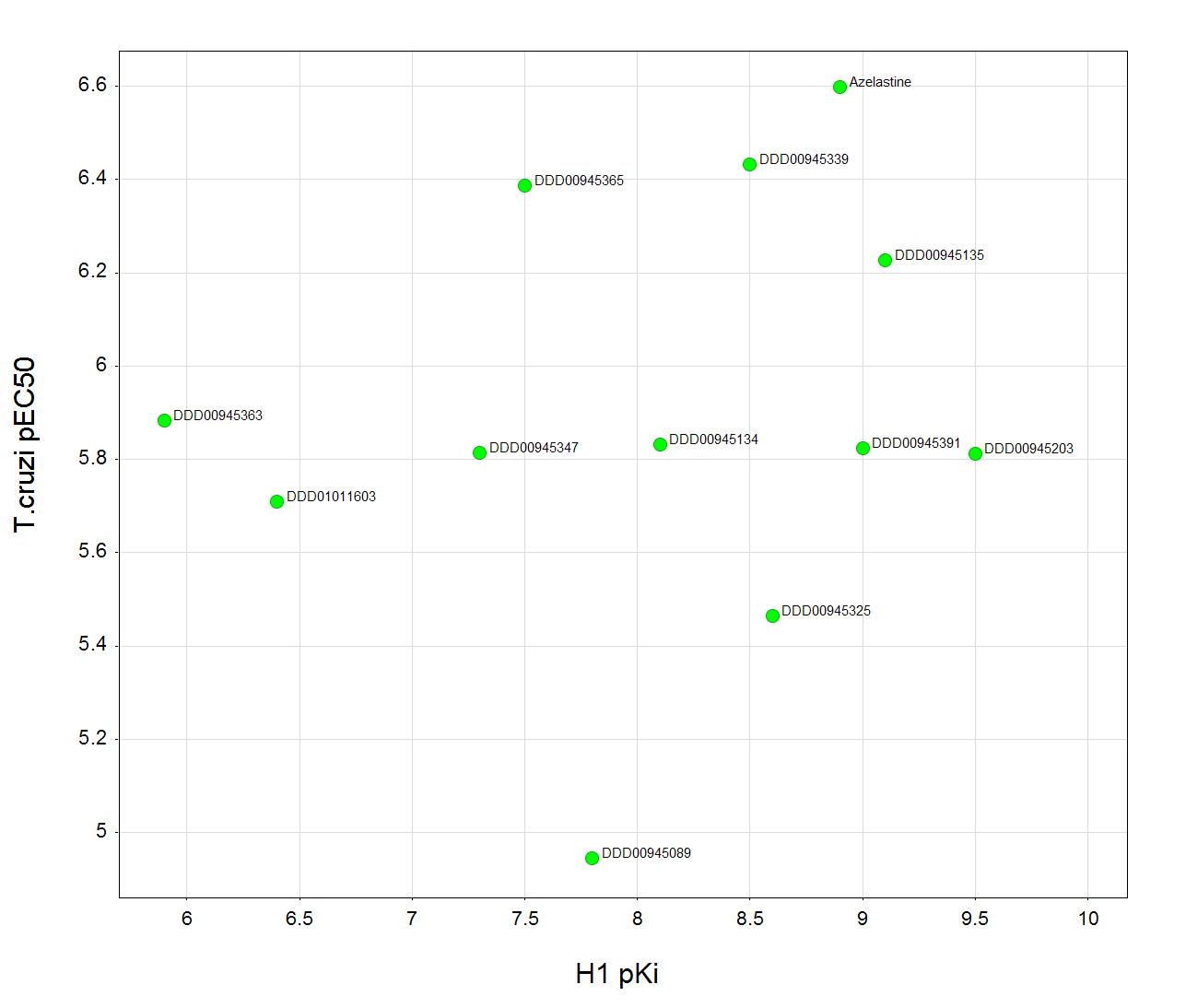
**

**Table B Azelastine analogues**

| **Structure** | **DDD Code** |
| --- | --- |
| \| C_22_H_24_ClN_3_O \| \| --- \| | Azelastine |
|    \| C_54_H_69_Cl_2_N_9_O_2_ \| \| --- \| | DDD00945089 |
| \| C_29_H_35_ClF_3_N_5_O_4_ \| \| --- \| | DDD00945134 |
| \| C_24_H_26_ClF_3_N_4_O_4_ \| \| --- \| | DDD00945135 |
| \| C_22_H_25_ClN_4_O_2_ \| \| --- \| | DDD00945203 |
| \| C_27_H_31_ClF_3_N_5_O_4_ \| \| --- \| | DDD00945325 |
| \| C_26_H_29_ClF_3_N_5_O_4_ \| \| --- \| | DDD00945339 |
|    \| C_26_H_33_Cl_2_N_5_O_3_ \| \| --- \| | DDD00945347 |
|    \| C_28_H_36_Cl_2_N_4_O_2_ \| \| --- \| | DDD00945363 |
|    \| C_26_H_34_Cl_2_N_4_O_2_ \| \| --- \| | DDD00945365 |
| \| C_28_H_34_ClF_3_N_4_O_6_ \| \| --- \| | DDD00945391 |
| \| C_25_H_31_N_3_O \| \| --- \| | DDD01011603 |
